# Corpus of Mandarin Child Language: a preliminary study on the acquisition of semantic content categories in Mandarin-speaking preschoolers

**DOI:** 10.3389/fpsyg.2023.1234525

**Published:** 2023-11-10

**Authors:** Tempo Po-Yi Tang, Dustin Kai-Yan Lau, Man-Tak Leung

**Affiliations:** Department of Chinese and Bilingual Studies, The Hong Kong Polytechnic University, Kowloon, Hong Kong SAR, China

**Keywords:** semantic content category, language corpus, Mandarin-speaking children, cognitive and syntactic complexity, acquisition

## Abstract

In studying language acquisition in children, sizable research studies have been focusing on the investigation of form and lexical semantics. This study aims to establish a child language database annotated both syntactically with part of speech and semantically with semantic content category to supplement the study of child language acquisition in the semantic domain beyond lexical level. The Corpus of Mandarin Child Language (CMCL) that documented the production of different semantic content categories by Mandarin-speaking children was established. Naturalistic language samples of 82 native Mandarin-speaking children aged 25–60 months, divided into three age groups, were obtained. The corresponding semantic content categories coded in each utterance were tagged according to previous studies, in addition to the annotations of part of speech. MLU and lexical diversity were examined and the usage and acquisition of different semantic content categories were also analyzed. The results regarding syntactic complexity and lexical diversity replicated the typical language acquisition pattern from previous studies, which supported the validity of the data obtained in the CMCL. To investigate the trajectory of acquisition of various semantic content categories by age, a 90% acquisition criterion was used. Our findings regarding the acquisition order of semantic content category were basically in line with previous studies in general, with some minor differences. This acquisition order observed is largely explained by the cognitive and syntactic complexity associated with the semantic content category, with additional influence from language specific properties and cultural specific factors of Mandarin. In addition, with the tags in both part-of-speech and semantic content category, the CMCL potentially provides a platform for examining the form-content interface in early child language acquisition, which also implies significantly on the theoretical and clinical ground.

## Introduction

Language is a unique and conventional tool for human to communicate effectively. Children acquire language rapidly in their early ages. They gain the ability to communicate using words, phrases and gradually more complex sentences to express various meanings efficiently. The underlying processes by which children achieve this remarkable language acquisition has long been a hot topic for linguists, psycholinguists and neurolinguists. To understand the role of linguistic knowledge in language acquisition, Bloom and Lahey (1978) proposed that content, form and use are three basic dimensions of language. In general, language content concerns the meaning of language, typically referred as the topics and ideas that are encoded in the linguistic messages. Language form concerns the structure of language and represents the rules that govern how particular language features are arranged. Language use, on the other hand, refers to the goals for communication, as well as how people express the goals in various contexts.

Existing research in child language acquisition had focused primarily on the use of language forms. In particular, large number of studies have been conducted to investigate the sentence length in young children. The pioneer study by Brown (1973) found that children’s mean length of utterance in morphemes (MLU) increased with age, and the children progressed through stages of grammatical development with increase in utterance length. Besides, the emergence of different parts of speech in the early vocabulary of young children has also been examined extensively (e.g., Lieven et al., 1992; Bleses et al., 2008). Likewise, sizable number of studies have also been conducted to investigate the acquisition of various morphological and syntactic constructions. For instance, early studies on child language (e.g., Brown, 1973; de Villiers and de Villiers, 1973) have established that different English inflections are acquired in a consistent order among young children. Similarly, the acquisition of various syntactic structures and categories was also investigated by Scarborough (1985). They found that children’s syntactic complexity and diversity increased with age, indicating that the older children produced more advanced and a larger variety of syntactic structures than the younger ones. In another study by Demuth and McCullough (2009), it was reported that preschool children acquired more complex sentence structures with auxiliary verbs gradually when they grew up. These studies contribute greatly to our understanding of child language acquisition from the perspective of language form.

In addition to the above, the importance of semantic contents in the study of child language has also been emphasized. Bowerman (1973) claimed that semantic but not syntactic knowledge should attribute the early sentence expressions in children. Besides, Brown (1973) also proposed that young children start to combine words semantically rather than grammatically when they have acquired around 50 words in an early stage. One example is that young children may produce telegraphic speech like “daddy chair” to express the content of possession, in which the grammatical possessive marker “s” is missing. Bloom (1970) further argued that children use meaning relations between words to discover syntactic rules. It was suggested that they first acquire the syntax of simple sentences to express a core of simple meanings, and subsequently the syntax of complex sentences in order to express the relational meanings between different propositions conveyed by the simple sentences. More recently, a growing body of literature on the integral role of semantic knowledge in child syntax acquisition has also been reported (Pérez-Leroux et al., 2008; Mok and Kipka, 2009). Given the importance mentioned above, it will be invaluable to examine the role of semantic content in the acquisition of early expressions in children.

In view of the above, some studies attempted to investigate lexical semantics in the acquisition of early vocabulary in children. For instance, Jiménez et al. (2021) investigated the lexical profiles of typically developing children by adopting the MacArthur-Bates Communicative Development Inventories (CDI; Fenson et al., 1993) to assess the production of twenty-two semantic categories. Apart from the investigation of semantic categories, the impact of various semantic measures and semantic features in language acquisition was also examined. Stockman and Vaughn-Cooke (1992) suggested that the acquisition of locative words was influenced by the meaning and relational roles of the words, while Horvath et al. (2022) also reported the impact of manner/result features of verbs on their acquisition in children. The results of these studies provided an alternative window for us to understand the acquisition process in young children in addition to the study of the acquisition of surface form. Nevertheless, existing studies investigating the semantic domains in language acquisition primarily focus on lexical semantics in terms of semantic categories and different semantic features. Only a limited number of studies attempted to go beyond the lexical level and examine the acquisition of various semantic contents directly (e.g., Green, 1975; Freedman and Carpenter, 1976; Stockman and Vaughn-Cooke, 1986; Bloom, 1991).

Mandarin, the official language spoken in China, is the language with the most native speakers in the world. It is of particular importance to understand how young children acquire Mandarin. A vast amount of acquisition studies on Mandarin-speaking young children have also been conducted. Regarding studies on language form, Brown’s (1973) findings on the increase of sentence length across ages were replicated in Chinese-speaking children (e.g., Cheung, 1998; Klee et al., 2004; Jin and Jin, 2008; Wu, 2020). Besides, Huang et al. (2022) investigated the early grammatical markings in toddlers who speak Mandarin. It was found that the acquisition trajectories of these markers were different from those in English and other languages, supporting the linguistic specificity in the acquisition of morphology. In addition, it was also indicated that more perceptually salient and obligatory markers were acquired earlier. On the other side, studies have also been conducted to investigate the acquisition of various syntactic structures including SVO utterances and *ba* constructions (Hsu, 2014) as well as relative clauses (Chen and Shirai, 2015). As for lexical semantic domains, Hao et al. (2008) and Tardif et al. (2009) reported the acquisition of different semantic categories in the early vocabulary inventory of young Mandarin-speaking children using parent-report questionnaire. On the other hand, Ma et al. (2009) also demonstrated the effect of the semantic measure of imageability on the acquisition of verb in both Chinese and English. Likewise, semantic features of temporal remoteness and specificity on the acquisition of different temporal-marker categories were also reported (Tang et al., 2021).

To this end, the acquisition of semantic content beyond lexical level in Mandarin remains relatively unknown. Given the importance of semantic content in young children’s language acquisition (e.g., Brown, 1973; Bloom, 1991), the current study aimed to investigate the acquisition of various semantic content categories (Lahey, 1988) in Mandarin-speaking children, in order to fill the research gap by contributing additional information from the semantic perspective. A database annotated both syntactically and semantically was established to investigate the above issues. In addition, the potential use of the database in examining the interaction between language form and content in the acquisition process was also explored. A brief introduction to various semantic content categories, followed by a review of previous studies on the acquisition of semantic content categories are given below.

### Semantic content category in young children

Semantic content is one component of language that concerns with the meaning of information, including the concepts and themes that language expresses. Young children typically express contents that reflect their immediate environment and experiences (Bloom and Lahey, 1978). A wave of crosslinguistic studies in the 1970s has proposed that young children, regardless of their native language, exhibit common topics and ideas in their early expressions (e.g., Bowerman, 1973; Brown, 1973). Similarly, Slobin (1985) crosslinguistic acquisition studies also echoed the restricted set of meanings expressed by young children. Other studies also reported cross-culturally common aspects of semantic relations (Raybeck and Herrmann, 1990) and antonymic meanings (Raybeck and Herrmann, 1996). As such, Lahey (1988) identified 21 semantic content categories in the production of young children according to the general kinds of meaning they share. The semantic content categories are listed in Table 1 with examples.

**TABLE 1:** List of semantic content categories with examples, modified from Lahey (1988).

No.	Semantic content category	Abbreviation	Example with Chinese translation
1.	*Existence*	exist	汽车 “car”
2.	*Recurrence*	recur	再 “again”
3.	*Nonexistence*	nonE	没有 “none”
4.	*Rejection*	rej	别 “do not”
5.	*Denial*	den	不是 “is not”
6.	*Attribution*	attri	大的 “big”
7.	*Possession*	poss	我的 “My”
8.	*Locative action*	locA	去 “go”
9.	*Action 12.*	act	睡觉 “sleep”
10.	*Locative state*	locS	上面 “above”
11.	*State*	state	饿 “hungry,” 黑暗 “dark”
12.	*Quantity*	quan	许多 “many”
13.	*Notice*	noti	看到 (他在跑)”see (him running)”
14.	*Dative*	dat	跑你 (一支笔) “give you (a pen)”
15.	*Additive*	add	…和… “and”
16.	*Temporal*	temp	刚刚 “just”
17.	*Causal*	caus	因为… “because”
18.	*Adversative*	adver	但是… “but”
19.	*Epistemic*	epis	怀疑 (是他做的) “suspect (it is done by him)”
20.	*Specification*	spec	穿红衣服的男孩 “the boy who wears in red”
21	*Communication*	com	告诉 (我一个秘密)“tell (me a secret)”

Adopting the framework of semantic content category, Brown (1973) analyzed the first fifty words produced by children speaking English, Finnish and Spanish. Despite differences in the specific words used across languages, the semantic content categories were remarkably similar across different languages. Besides, the studies of Stockman and Vaughn-Cooke (1982, 1986) also provided evidence on the equivalent set of semantic content categories among young African American children, for which forms are mapped onto the same underlying concepts, regardless of culture and whether standard forms are used.

To the best of our knowledge, no previous studies have been done on the acquisition of semantic content categories by Mandarin-speaking young children. Despite a few previous studies have suggested cultural influences in particular meanings like emotion (e.g., Herrmann and Raybeck, 1981; Russell, 1991), the major studies appeared to indicate that the above-mentioned semantic content categories were found in early child language in general (e.g., Bowerman, 1973; Brown, 1973). Therefore, the current study adopted this specific set of semantic content categories and investigated their acquisition in Mandarin accordingly.

### Acquisition of semantic content categories

There are a few studies examining the acquisition of semantic content categories by English-speaking young children. The study conducted by Stockman and Vaughn-Cooke (1986) investigated the order of acquisition of various semantic content categories with twenty-two standard and non-standard English speakers aged 18–54 months. Results indicated that *existence* and *action* were the earliest acquired semantic content categories and were the only ones productively produced by every child. After that, the semantic content categories of *locative action*, *state*, *locative state*, *negation*, *possession*, *attribution*, *notice*, and *recurrence* also emerged, whereas semantic content categories of *additive*, *causal*, and *epistemic* were acquired last. This study did not only present preliminary acquisition trends of various semantic content categories in young children, but also indicated that both standard and non-standard speakers shared the same semantic base in early language although the forms used were different.

Bloom (1991) also carried out a series of longitudinal studies to investigate the acquisition of semantic content categories of children aged 24–38 months by using naturalistic language samples. The expressions of various semantic content categories related to early sentences and complex sentences were examined, respectively. First, looking at the earliest appeared semantic content categories of simple sentences, contents *of existence*, *non-existence*, and *recurrence* were found to emerge before *action*, *locative state*, and *locative action*. Specifically, among the semantic content categories related to verb, it was found that *action* emerged first, followed by *state* and then *locative action*. Finally, *dative* and *specification* came along after the above basic verb relations. The emergence of *possessive* and *attribution*, on the other hand, varied among the participants. Apart from the above, a specific order of acquisition for semantic content categories of negation was also suggested, with *non-existence* emerged before *rejection*, while *denial* emerged last.

Next, eight semantic content categories associated with complex sentences were observed in the samples, including *additive*, *temporal*, *causal*, *adversative*, *specification*, *epistemic*, *notice*, and *communication.* Among those involving the use of conjunctions, the acquisition followed the sequence: *additive > temporal > causal > adversative*. On the other side, *epistemic*, *notice*, and *communication* were observed to emerge after the above, while the content category of *specification* was infrequent in the language samples of all participants.

### Factors affecting the acquisition of semantic content categories

The above acquisition order of semantic content category allows us to understand how content affects language acquisition in young children. This relative order of acquisition is affected by two major factors in general: syntactic complexity and cognitive complexity (Bloom, 1991). From the syntactic view, semantic content categories related to complex sentences in general appeared later than those associated with simple sentences (e.g., *existence* and *possessive* emerged before *causal* and *specification)*. Specifically, among the semantic content categories expressed using verbs, utterances expressing *locative action*, which usually involves the description of places, are syntactically more complex than descriptions of the motion or location alone in utterances expressing *action* or *locative state*. Likewise, the expressions of *dative* content, which describe two different kinds of relations between recipients and affected objects, are also syntactically more complex than utterances expressing only *action* and *locative action*, and thus emerged later. Regarding semantic content categories associated with complex sentences, those involving the use of syntactic structures with conjunctions (i.e., *additive*, *temporal*, *causal*, *adversative*) generally appeared earlier than those related to more complex complementation (i.e., *epistemic*, *notice*, *communication).* Semantic content category related to relativization (i.e., *specification*) is regarded as more complex syntactically and thus emerged last.

Cognitive complexity was suggested to be another factor accounting for the acquisition order of various semantic content categories according to Bloom (1991). Children learn that objects exist by acting in ways that make them disappear and recur in the sensorimotor period (Piaget, 2000). This relatively simple object permanence explains the earliest emergence of *existence*, *non-existence*, and *recurrence*. Then, children learn that objects can be acted upon and located in space, as reflected in the emergence of verb relations including *action*, *state*, *locative state*, and *locative action*, which came immediately after the previous functional relations. In particular, *locative action* representing the dynamic concept of the movement of objects between two places, usually involving source, path and goal, is more complex cognitively and appears later than *action* and *locative state*, which are static in nature. Likewise, the expression of *attribution* requires discrimination among similar objects, and thus higher-level cognitive processes of categorization and seriation, whereas *action* only involves comparatively simpler sensorimotor schemas in its production. Therefore, *action* preceded *attribution* in the acquisition. Finally, the involvement of a cognitively more complex symbolic referent in *denial* may also partially account for its later acquisition than *non-existence* and *rejection*, which negate more concrete objects or actions, respectively.

The principle of cognitive complexity also applies to the acquisition among semantic content categories associated with complex sentences. Bloom (1991) suggested that there is a progressive increase in the cognitive complexity among the acquisition of *additive*, *temporal*, *causal*, and *adversative*. Firstly, *additive* simply represents the indefinite joining of two events and is regarded as relatively simple. *Temporal* conveys the relations of two or more events with designated temporal sequences, while *causal* relations express the antecedent and consequence of events on top of the additive and temporal concepts. *Adversative* appears to be the most complex one, which involves additive, temporal and sometimes, causal concepts, as well as expressing the new meaning of opposition which involves the cognitive process of comparing and contrasting. As a result, the cumulative cognitive complexity among these semantic content categories tends to explain their corresponding status in the acquisition process.

To achieve the goal of investigating the acquisition of semantic content categories in Mandarin-speaking children, the acquisition trajectory reported in English will be used as a reference. In light of the above factors of syntactic and cognitive complexity, it is predicted that the acquisition of semantic content categories among Mandarin-speaking children basically follows the trajectory reported in English, with some minor differences due to the syntactic properties of Mandarin and cultural specific factors.

### Methodological concerns

To study child language acquisition, language sample analysis (LSA) has been used extensively. LSA based on the spontaneous speech of children was found to have the advantage of being an ecologically valid and authentic method of assessment (Owens, 2010) and the language samples are suggested to be usually reflecting a more naturalistic and representative picture of the child’s language over the standardized assessments (Evans and Craig, 1992). Extensive research studies have employed LSA to examine the acquisition of different linguistic parameters, such as early lexical development (e.g., Liu, 2007; Stoll et al., 2012), early grammatical development (e.g., Lee and Wong, 1998), syntactic complexity (e.g., Diessel, 2004; Lu, 2009; Deng et al., 2018), morphology (e.g., Maslen et al., 2004; Jia and Fuse, 2007), and discourse relationship (e.g., Zhou and Xue, 2015).

Language corpora of transcribed and usually annotated language samples, on the other hand, are also essential for the study of child language acquisition. According to Biber et al. (1998), using corpus to study language is empirical as a large collection of samples from real-life situations is utilized. Availability of computer analytical techniques also speeds up and enhances various quantitative and qualitative analyses of samples in different language aspects. Quantitatively, the distribution and properties of different structures can be calculated for types, tokens and percentages whereas qualitatively, both target and non-target structures can also be investigated (Deng and Yip, 2018). In addition, the corpora can be used for multiple times and be available to others if made publicly. Therefore, in the current study, both LSA and language corpora were adopted to document the production of Mandarin-speaking children in both syntactic and semantic domains.

Among currently available cross-sectional Mandarin corpora, many of them included good sample size and naturalistic procedures for collecting children’s language samples (e.g., Li and Zhou, 2008; Li and Zhou, 2015), which provide important resources for studying child language acquisition. Nevertheless, these corpora were only annotated syntactically and none of them possessed annotations on semantic content category. Most importantly, it seems that expressions of certain semantic content categories were not specifically elicited in the language samples of the existing databases, which did not allow the investigation from the semantic perspective.

As discussed above, the semantic content of language also contributes potential values in language acquisition studies. While *form* and *content* are equally important domains of language, semantically annotated data provides an alternative window for the investigation of the acquisition process in child language. Sagae et al. (2010) also suggested that adding semantic information in the corpora should be a future direction in language acquisition studies. Therefore, it would be of great importance to establish a child language database with corresponding annotations of semantic content categories on the samples collected. To avoid the issue that children’s language productions were dictated dominantly by the topics initiated by the interviewers, a standardized protocol was used to minimize the influence of different interviewers, as well as to create equal opportunities across children to elicit all the intended semantic content categories specifically. Besides, the typical measures of sentence complexity and lexical diversity in language acquisition were also calculated, to ensure the representativeness of the language samples collected.

### The current study

In short, while the majority of previous studies on child language acquisition had been focusing on the study of form or lexical semantics, relatively few studies examine the semantic domain beyond lexical level in early language acquisition. In particular, there is currently no study investigating the acquisition of semantic content categories among Mandarin-speaking children. As a result, the current study aimed to establish the Corpus of Mandarin Child Language (CMCL), a corpus annotated with both part-of-speech and semantic content category, and adopted language sample analysis by extracting data from the corpus, for the study of the acquisition of semantic content categories in Mandarin-speaking children. The following research questions were addressed in the study:

1. What is the acquisition trajectory of various semantic content categories among Mandarin-speaking children?
2. What are the similarity and difference in the acquisition trajectories of semantic content category between Mandarin-speaking and English-speaking children?

To address the concern on the applicability of the language data in CMCL, the measures on sentence complexity and lexical diversity of the children’s productions were also obtained. It was expected that the age differences in these traditional measures from previous studies would be replicated in the current study. On the other side, the acquisition trajectory of semantic content category among Mandarin-speaking children was also predicted to follow the sequences suggested in Bloom (1991) in general. Syntactically, semantic content categories related to simple sentences would appear earlier than those related to complex sentences. Similarly, semantic content categories associated with lower cognitive complexity would also precede those cognitively more complex ones in the acquisition. Nevertheless, subtle differences might be expected, due to the linguistic and cultural issues which are specific to Mandarin. This current study not only sheds new light on the understanding of language acquisition in the semantic domain, but also allows the investigation of the form-content interface and contributes to validating different theories in the early language of children. Moreover, clinical implications for the assessment and intervention of children with language disorders are also provided.

## Materials and methods

### Participants

The Corpus of Mandarin Child Language (CMCL) is established with 82 native Mandarin-speaking children aged 25–60 months (48 boys and 34 girls) recruited from early education centers and kindergartens in Shenzhen and Guangzhou, China. According to their caregivers, none had any sensory or intellectual disabilities or language problems. All participants were divided into three age groups by 1-year intervals (i.e., 25–36, 37–48, and 49–60 months). Information on the subjects is shown in Table 2.

**TABLE 2:** Subject information of CMCL (*N* = 82).

Age group	*n*	Age range (months)	Mean age (months)	*n* male	*n* female
1	19	25–36	30	13	6
2	41	37–48	42	24	17
3	22	49–60	55	11	11

### Language sample collection and transcription

Participants’ language samples were elicited individually by speech therapists, speech therapy students and research assistants who had received prior training. First, a warm-up period with a doll set or train set was used for rapport building before conducting the three tasks of taking actual language samples (i.e., free play with toys, storytelling with pictures, and conversations). Semi-spontaneous speech of each child was then collected through one-to-one interactions with the examiner on three tasks following the standardized procedures in CMCL. Identical sets of toys, including a cooking set, food, utensils, puppets and a mystery bag, were provided during each 20-min freeplay session. Each child was encouraged to play and communicate with the examiner. Importantly, the play was specially designed to provide a scenario for eliciting different semantic content categories. Open-ended questions, parallel play and parallel talk were employed to facilitate the children’s own language production. A color Cookie Theft picture (Goodglass and Kaplan, 1972) and a set of four card stories were then provided to elicit each child’s narrative speech, which lasted about 5 min. Finally, the examiner initiated a 5-min talk about daily life according to the child’s interest and experience. A Peppa Pig storybook was also presented to provide topics for the chat (e.g., picnicking and favorite cartoon characters).

All sessions were audio- and video-recorded and all utterances produced by both the participants and interviewers were transcribed orthographically. Intonation contours, pauses of more than 2 s, and speaker turns were used to determine the utterance boundaries (Klee and Fitzgerald, 1985). All the utterances produced by the participants were then analyzed by the trained speech therapy students and research assistants, but short responses, self-repetitions, unintelligible and incomplete utterances which did not reflect the children’s own language ability, were not analyzed (Crystal et al., 1989). The physical context was provided by the descriptions of the events and the actions of participants, while the linguistic context was shown with the examiner’s utterances.

To ensure transcription accuracy, the transcribers, all holding a degree in linguistics, were extensively trained prior to the beginning of the study. Language samples obtained from a pilot study were transcribed together and any discrepancies were resolved through discussion. A manual with a detailed set of transcription guidelines was eventually agreed. The audio- and video-taped samples also allowed multiple viewing when necessary. Finally, a number of transcripts independently transcribed by each of the transcribers were compared using the RELY function in CLAN (Codes for Human Analysis of Transcripts) to check for transcription consistency. The percentage of the overall match of words between the transcribers was 95.4%.

### Database

All the above data were imported to the software Filemaker Pro which provides an interface to display the content of CMCL. The orthographic transcription of child utterances formed the first main layer in the database. Every single utterance produced by the child and interviewer was represented as one entry. Next, words within each utterance were identified according to the principles of boundedness, expandability, and versatility proposed by Zhu (1982). For each word, tags on phonological form using *pinyin*, English translation and part of speech were added with reference to the MOR databank provided by CHILDES (MacWhinney, 2000). Words that could not be found in the “zho” dictionary in the MOR databank were identified and added manually. The newly added words mainly included names of specific people, places, cartoon programmes and cartoon characters. Specific food items (e.g., 甜甜圈 “donut”) and kitchen utensils (e.g., 蒸锅 “steamer”), as well as reduplication of words [e.g., 鸡鸡 “chicken” (noun); 神秘秘 “mysterious” (adjective)] were also added.

Finally, the utterances were annotated with 21 semantic content categories based on the descriptions in Lahey (1988), to form the third semantic tier. Assignment of semantic content categories was done by trained speech therapist students. Notably, a particular semantic content category can be encoded with different syntactic forms [e.g., content of *temporal* can be expressed with aspect marker, temporal adverb and temporal noun by Chinese-speaking young children (Zhou, 2004; Tse et al., 2012)], whereas a particular form can also be used to encode different semantic content categories (e.g., the phrasal structure 公园的树木 represents location, 红色的杯子 represents attribution, while 爸爸的头发 represents possession). In this study, the semantic content categories associated with different utterances were annotated based on the participants’ intended meaning determined according to the corresponding physical and language contexts shown in the video. After all the semantic coding had been done, 10 percent of the speech samples were randomly selected and independently coded by a second rater to develop inter-rater reliability. A relatively high agreement of 93.0% of semantic content category coding was achieved between raters.

### Data analysis

Data were extracted from the CMCL for further analysis. Each child’s major utterances except the deviant ones were used to calculate the mean length of utterance in word (MLU), following Zhu (1982) and Cheung (1998) procedures. Traditional measures on lexical diversity including number of different words produced (NDW), total number of words produced (TNW), type-token ratio (TTR) and number of different open/closed class words were examined. In addition, vocD (Malvern and Richards, 2002) was also calculated automatically by running the MOR command in CLAN. Finally, the usage and acquisition of different semantic content categories were also analyzed.

## Results

There are altogether 13, 630 utterances produced by 82 children from the age of 25–60 months (mean age = 43 months, 48 boys and 34 girls). Among all the child utterances, only 10,643 were analyzed in CMCL, while the remaining 2,987 utterances (22% of all child utterances) including short response, repetition, incomplete or unintelligible utterances were not analyzed. Results on utterance length and lexical diversity, as well as the usage and acquisition of different semantic content categories were illustrated as follow.

### Utterance length and lexical diversity

Table 3 presents the descriptive statistic information of children’s MLU, NDW, TNW, TTR, and vocD across different age groups. To investigate the effect of age on sentence length, a one-way analysis of variance (ANOVA) was conducted. Results showed a significant effect of age on MLU [*F*_(2,79)_ = 11.02, *p* < 0.001]. *Post-hoc* analyses using the Bonferroni’s test at a significance level of 0.05 indicated that the 2 year olds possessed lower MLU than the 3 year olds, but the difference between the 3 year olds and the 4 year olds was not significant.

**TABLE 3:** Different language measures of child utterances across age group.

	Age group 1			Age group 2			Age group 3		
	**Mean**	**S.D.**	**Range**	**Mean**	**S.D.**	**Range**	**Mean**	**S.D.**	**Range**
MLU	2.73	0.55	1.74–4.3	3.61	0.82	2.26–5.86	3.78	0.77	2.14–5.63
NDW	113.61	27.46	64–179	168.26	40.45	102–265	163.05	45.26	108–258
TNW	507.78	186.32	192–883	679.14	272.83	216–1446	537.09	249.7	277–1106
TTR	0.24	0.06	0.14–0.38	0.27	0.07	0.15–0.50	0.33	0.07	0.20–0.46
TNU	136.39	46.02	36–193	142.5	42.22	62–241	116.59	40.69	62–206
vocD	33.5	8.53	15.9–47.0	41.1	12	16.0–64.7	48.3	6.93	34.6–60.2

MLU, mean length of utterances in word; NDW, number of different words; TNW, total number of words; TTR, type token ratio; TNU, total number of utterances.

Regarding lexical diversity, a one-way ANOVA was conducted to analyze the effect of age on each of the measures of NDW, TNW, TTR and vocD. Results showed a significant age effect on NDW [*F*_(2,79)_ = 15.58, *p* < 0.001], TTR [*F*_(2,79)_ = 9.41, *p* < 0.001], and vocD [*F*_(2,79)_ = 18.35, *p* < 0.001]. *Post-hoc* Bonferroni’s test at a significance level of 0.05 revealed that the observed NDW was lower in the 2 year olds than the 3 year olds, while the 3 year olds also possessed lower TTR than the 4 year olds. On the other hand, lower vocD was also observed in both the 2 year olds and the 3 year olds when compared with their older counterparts, respectively. Pearson’s correlation was also conducted to investigate the relations between sentence length and various measures of lexical diversity. Table 4 presents the correlations among children’s MLU, NDW, TNW, TTR, and vocD. The results indicated that MLU is significantly correlated with NDW (*r* = 0.662, *p* < 0.01) and TTR (*r* = 0.662, *p* < 0.01), but not with vocD (*p* > 0.05).

**TABLE 4:** Correlation between children’s age, mean length of utterances, number of different words, total number of words, and type-token ratio.

Variables	1	2	3	4
Mean length of utterance in word	–			
Number of different words	0.662**	–		
Total number of words	0.509**	0.807**	–	
Type-token ratio	0.662**	1.0**	0.807**	–
vocD	0.144	0.145	−0.081	0.334**

**p < 0.01.

The number of different open class words (nouns, verbs and adjectives) and closed class words produced by children in each age group was shown in Table 5. Results of correlations (Pearson’s r), as shown in Table 6, indicated that children’s age is significantly correlated with number of different nouns produced (*r* = 0.415, *p* < 0.01), number of different verbs produced (*r* = 0.282, *p* < 0.01), and number of different adjectives produced (*r* = 0.451, *p* < 0.01), but not with number of different closed class words produced (*p* > 0.05).

**TABLE 5:** Average number of different lexical items across age group.

	Age group 1		Age group 2		Age group 3	
	**Mean**	**S.D.**	**Mean**	**S.D.**	**Mean**	**S.D.**
Noun^∧^	28.28	10.86	47.5	12.67	47.77	15.57
Verb^∧^	21.78	7.12	34.05	9.86	32.36	9.51
Adjective^∧^	8.06	3.32	17.19	6.33	17.95	5.71
Closed class words^∧^	55.5	10.42	69.52	16.02	64.95	18.68

^∧^Number of different items in that particular lexical class.

**TABLE 6:** Correlation between children’s age and number of different lexical items.

Variables	1	2	3	4	5
Age	–				
Noun^∧^	0.415**	–			
Verb^∧^	0.282*	0.775**	–		
Adjective^∧^	0.451**	0.748**	0.729**	–	
Closed class words^∧^	0.103	0.792**	0.805**	0.727**	–

*p < 0.05.

**p < 0.01.

^∧^Number of utterances expressing that particular lexical item.

### Semantic content categories

Table 7 presents the average number of different semantic content categories across age groups. Results of correlations (Pearson’s r) indicated that the number of different semantic content categories is significantly correlated with age (*r* = 0.269, *p* < 0.05) and MLU (*r* = 0.332, *p* < 0.001). The average token count and the number of different semantic content categories per utterance produced by children in different age groups can also be found in Supplementary Tables 1, 2, respectively. It was noted that semantic content category of *specification* was absent in all age groups.

**TABLE 7:** Average number of different semantic content categories across age groups.

	Age group 1			Age group 2			Age group 3		
	**Mean**	**S.D.**	**Range**	**Mean**	**S.D.**	**Range**	**Mean**	**S.D.**	**Range**
Number of different semantic content categories	14.5	2.2	9–19	16.5	1.90	13–20	16.4	1.40	14–19

To investigate the acquisition trajectory of different concepts within certain linguistic domains among children, one common approach is the use of a 90% criterion (e.g., Brown, 1973; So and Dodd, 1995), in which a concept is considered acquired by the majority of children in a particular age group if 90% of the children in that age group demonstrate the correct usage of that concept in the elicitation experiment. For example, researchers have examined the order of morpheme acquisition by looking at the age at which 90% of the children were reported to produce the morphemes (e.g., Brown, 1973; de Villiers and de Villiers, 1973). Likewise, a 90% criterion has also been used in research documenting the acquisition trajectory of different phonemes among young children (e.g., So and Dodd, 1995; Zarifian et al., 2015; Crowe and McLeod, 2020). To investigate the age of acquisition for different semantic content categories, the 90% criterion was therefore adopted. A semantic content category was regarded as acquired by the particular age group if 90% of the participants in the group produced the semantic content category at least once in the sample collected. Table 8 summarizes the semantic content categories acquired by participants from 2 to 5 years old. Among the 2-year-old participants, nine semantic content categories were acquired (*existence*, *non-existence*, *reject*, *attribution*, *action*, *locative state*, *state*, *quantity*, *temporal*). Notably, the semantic content category of *reject* reached 90% occurrence in this age group but declined and did not meet the acquisition criterion in the two older age groups. Three more categories (*denial*, *possession*, *locative action*) were added to the inventory of the 3 year olds while another three categories (*dative*, *additive*, *causal*) were further acquired by the 4 year olds. Six semantic content categories, namely *recurrence*, *notice*, *adversative*, *epistemic*, *specification*, and *communication*, were not fully acquired even by the 5-year-old group.

**TABLE 8:** Occurrence of different semantic content categories by age group.

	2 year olds	3 year olds	4 year olds
Existence	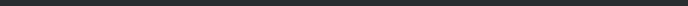		
Non-existence	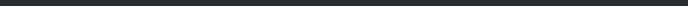		
Attribution	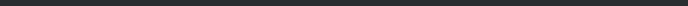		
Action	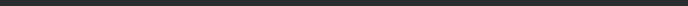		
Locative state	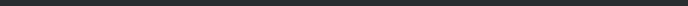		
State	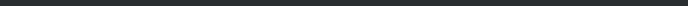		
Quantity	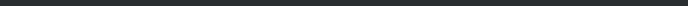		
Temporal	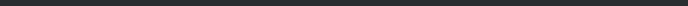		
Reject	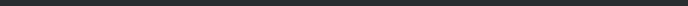		
Denial	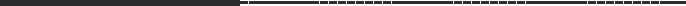		
Locative action	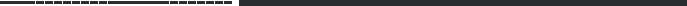		
Possession	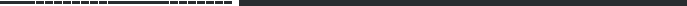		
Additive	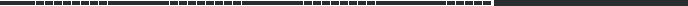		
Causal	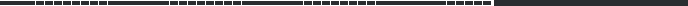		
Dative	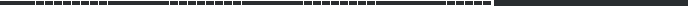		
Recurrence	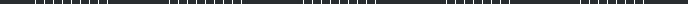		
Notice	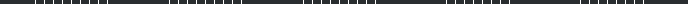		
Adversative	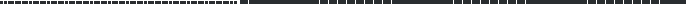		
Epistemic	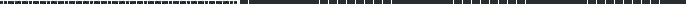		
Communication	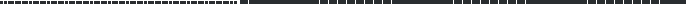		
Specification	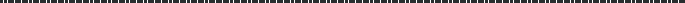		

A dotted line indicates that the semantic content category had <20% occurrence, a dashed line indicates 20%–89% occurrence, and a solid line indicates ≥90% occurrence.

## Discussion

The current study reported a database, the CMCL, that documents the language samples obtained from Mandarin-speaking children aged between 2 and 5 years old. The orthographically transcribed language samples were tagged with their corresponding part of speech and semantic content category. Before discussing the major focus of the acquisition pattern of semantic content categories, to ensure the representativeness of the content of the corpus, typical measures of utterance length and lexical diversity were obtained. Results indicated that the effect of age on sentence length and lexical diversity from previous studies were replicated. Details were elaborated in the following section.

### Utterance length and lexical diversity

In the current study, it was observed that shorter utterance length was associated with the younger age group in general. The increase in utterance length moving from the 2 year olds to the 3 year olds, as reflected in the MLU measure, is consistent with reports of previous studies on both English (Miller, 1981; Paul, 2000; Rice et al., 2010; Moyle et al., 2011) and Chinese children (Cheung, 1998; Klee et al., 2004; Jin and Jin, 2008; Wu, 2020). On the other hand, observations of the 4 year olds appeared to indicate a plateau in sentence length in this age group and replicated previous findings that MLU may be less sensitive in capturing the progress in language abilities of children beyond the earliest stage of language acquisition (e.g., Klee and Fitzgerald, 1985; Smith and Jackins, 2014).

Regarding the lexical diversity among children, NDW, especially for the open class words and TTR, appeared to reflect some age effects in the current study. However, it is noteworthy that the results also demonstrated positive correlations between NDW and TTR with MLU, but not between vocD and MLU. This echoed previous findings that suggested the possible confounds of NDW and TTR in representing the vocabulary skills of children, as both of them may be affected by sentence length (Klee, 1992). To this end, the current findings tended to support the notion that vocD is more suitable for measuring lexical diversity in the context of Mandarin (Zhang and Zhou, 2020). Besides, vocD also appeared to be a more sensitive measure in reflecting the progress of lexical diversity across age groups, as significant differences were found between both the 2-year-old and 3-year-old groups, as well as between the 3-year-old and 4-year-old groups. In sum, similar to the measure of sentence length, the increase in lexical diversity across age groups, as indicated in the measure of vocD, is also consistent with previous results with English-speaking (Miller, 1991; Owen and Leonard, 2002) and Chinese-speaking children (Klee et al., 2004; Jin and Jin, 2008; Wu et al., 2019; Zhang and Zhou, 2020). Generally, the results obtained from the CMCL replicated the typical language acquisition pattern from previous studies, which confirm that the procedures we applied in data collecting language samples provide a valid data source for studying language acquisition in young Mandarin-speaking children.

Open class words primarily convey the concrete content of the sentences whereas closed class words are usually more related to the grammatical aspects of sentences, and include relatively few members (Weber-Fox and Neville, 2001). It was observed that young children produced proportionally more open class words than closed class words in a previous study using parent questionnaires (Klintfors et al., 2009). In the current study, however, the productions of the open class words and closed class words were more comparable among the 2 year olds. The standardized elicitation probes used in the current study may be one possible reason that accounts for the different observations compared with previous literature. With the standardized elicitation probes that focused mainly on here and now contexts in the current study, the 2 year olds, who are more capable of producing contextualized expressions, may show higher tendency to produce only open class words targeted in the elicitation probes, and consequently produced only a limited number of them. On the other hand, closed class words include aspect markers, numerals, classifiers, determiners, pronouns, sentence final particles and prepositions. Some of them, like aspect markers and sentence final particles, are particularly prominent in Mandarin and are used extensively by young children (Erbaugh, 1992; Fang and Hengeveld, 2022). This may also lead to the production of apparently more closed class words among the 2 year old, resulting in a relatively even ratio between the number of open and closed class words observed.

The results from the current study indicated consistent findings on the traditional measures like MLU and vocD in the language acquisition of Mandarin-speaking children, thus confirming the representativeness of the corpus. In the following, we discussed the findings of our major focus regarding the acquisition of semantic content category in Mandarin-speaking children.

### Acquisition of semantic content categories and the underlying factors

Given the importance of the semantic domain in language acquisition mentioned before, the usage and acquisition of various semantic content categories were investigated to supplement the study of child language acquisition from the semantic perspective.

Results showed that the number of different semantic content categories increased with both age and sentence length. The increase in the unique semantic content categories thus offers a glimpse into the language acquisition of typically developing children from the semantic perspective along with the syntactic analysis, providing a more comprehensive picture of language acquisition. Moreover, by using the 90% criterion, the acquisition pattern of different semantic content categories in Mandarin-speaking young children were also investigated. Among the 21 semantic content categories, nine of them, namely *existence*, *non-existence*, *reject*, *attribution*, *action*, *locative state*, *state*, *quantity*, and *temporal*, were acquired early by the 2-year-old group. The early acquisition of these semantic content categories, except *temporal*, is generally coherent with previous studies (Stockman and Vaughn-Cooke, 1986; Bloom, 1991). The later acquisition of *temporal* content reported in Bloom (1991) may probably be due to their investigation of the temporal content related to the use of conjunctions only in that particular study, while aspect markers, which occur in Mandarin-speaking children as young as 18 months (Zhou, 2004), were included in our study of the temporal content category. Besides, according to Hao et al. (2008), Mandarin-speaking children aged 17–30 months experience a substantial vocabulary growth, and are capable in expressing vocabulary of objects, people, places and actions, as well as using some quantifiers, descriptive words, and words about time. By relating these vocabularies to their corresponding semantic content category (e.g., content of *existence* can be expressed with objects and people; content of *locative state* can be expressed with place; content of *attribution* and *state* can be expressed with descriptive words; content of *action* can be expressed by action words; content of *quantity* can be expressed by quantifier; content of *temporal* can be expressed by time words and aspect markers), it is found that the acquisition trajectory on semantic content categories in our study generally replicates previous research findings.

Adopting Bloom’s (1991) framework, the early acquisition of the abovementioned semantic content categories can be explained by the relatively simple cognitive complexity of the concept being coded. Johnston (1985) suggested that conceptual development is the prerequisite for semantic growth, while Slobin (1973) also proposed that complexity of the concepts affects the acquisition of linguistic terms, in which more abstract and complex ideas are acquired later. As a result, the cognitive complexity of the underlying concepts inevitably affects children’s semantic representations, and thus the acquisition of various semantic content categories. Specifically, acquisition of the semantic content categories of *existence*, *non-existence*, and *action* require the sensorimotor knowledge related to object permanence and should therefore be acquired in the sensorimotor period before age of two (Piaget, 2000). In addition, concepts of objects and events are perceptually easy as concrete referents are usually involved. Lahey (1988) also claimed that young children have possessed the basic knowledge on objects and events at an early age. Among the earliest acquired semantic content categories, *existence* and *non-existence* mainly represent objects, people and events; *locative state* is related to different locations; *action* conveys the meaning of motion which does not involve changes of location. Besides, *reject*, *attribution*, *state*, *quantity*, and *temporal* mostly serve to provide additional information of the object, action and event. The mapping between this concrete sensorimotor and cognitively simple knowledge of single object and event with words can therefore be established at an early age, and are reflected in their naturalistic speech expression.

Alternatively, the acquisition of these semantic content categories may also be related to linguistic factors. As children’s early vocabularies contain proportionally more open class words than closed class ones (Klintfors et al., 2009), it is not surprised to observe that some of the above semantic content categories which are mostly coded using open class words (e.g., *existence*, *action*, *attribution)* were acquired early. Furthermore, most of these semantic content categories can be coded with simple syntactic units, such as single words (e.g., *existence*, *non-existence*, *attribution*, *action*, *locative state*, *state*), simple noun phrase [e.g., “一个” (numeral + classifier) to code *quantity*] and simple verb-phrase [e.g., “吃了” (verb + aspect marker) to code *temporal*]. In line with Bloom’s (1991) proposal on the effect of syntactic complexity, semantic content categories related to simple sentences were therefore acquired first.

Moving on to the 3-year-old group, three more semantic content categories, namely *denial*, *locative action*, and *possession* were subsequently acquired. The emergence of these semantic content categories also followed the acquisition sequence reported in Stockman and Vaughn-Cooke (1986) and Bloom (1991) in general. The above acquisition is again considered to be related to the cognitive and syntactic complexity. Firstly, children progressively acquired the simple relations between objects and events on top of the knowledge in single objects and events (Lahey, 1988). To this end, the content of *denial* which negates attribute, identity and state of events (Chang, 1992), and the content of *locative action*, which expresses the change in locations including the source, goal and/or path, are used to represent the relations between objects and events. Similarly, the content of *possession* also expresses the relations between the owner or possessor and the entity. Acquisition of these semantic content categories representing the simple relational knowledge between objects and events tended to involve higher demand in cognitive processing, and therefore these semantic content categories emerged later than those acquired by the 2-year-old group.

The syntactic complexity associated with these semantic content categories may also play a role in the acquisition. To express the content of *possession* in Mandarin, it is proposed that the genitive marker “*de*” is the most common linguistic device being used (Shi and Zhou, 2018). Li (2004) also reported that the comprehensive use of possessive expressions with “*de*” was acquired by age of three, which appear to be in line with our observations on the acquisition of *possession*. Meanwhile, this grammatical marking “*de*” is often regarded as a bigger challenge than the content words in the acquisition by young children (Huang et al., 2022). On the other side, the content of *locative action* may also utilize co-verbs or prepositional phrases to encode both manner and path of the motion in Mandarin (Slobin, 2004). The unique thematic roles associated with different prepositions in Chinese may further impose difficulties in the acquisition of the corresponding constructions (Lau et al., 2023). These specific language properties of Mandarin thus constitute a higher syntactic complexity in encoding both *possession* and *locative action*, and account for their acquisition beyond 2 year old in Mandarin-speaking children.

Next, the 4-year-old group further acquired the semantic content categories of *additive*, *causal*, and *dative*. This again replicates the acquisition order reported in both Stockman and Vaughn-Cooke (1986) and Bloom (1991). The acquisition trend in this age group can also be explained according to the cognitive and syntactic complexity associated with the semantic content categories. First, both additive and causal represent specific relations of two events or states and should be cognitively more complex that those representing only one event. Besides, the content of causal additionally indicates a dependency between the events and gives the reason or result of the events, thus requiring more cognitive resources to process (Bloom, 1991). The above contributes an initial explanation for the acquisitions of additive and causal in this group of children.

On the other hand, acquisition of *dative* seems to be more related to the syntactic complexity. The coding of *dative* in Mandarin usually involves the use of different syntactic structures and prepositions with different themes [e.g., “送花给妹妹” (verb + noun + preposition + noun)] (Details about different syntactic structures and use of prepositions are elaborated in later section). As mentioned before, Chinese prepositions are usually associated with specific thematic roles, which impose additional challenges on the syntax constructions (Lau et al., 2023). As a result, a more complex syntactic structure entailing the relations between recipients and the affected objects is usually involved with *dative* expressions. Similarly, the coding of *additive* and *causal* contents also requires the conjoining of phrases or clauses with conjunctions [e.g., “面包和鸡蛋” (noun + conjunction + noun) to code *additiv*e; “生病就看医生” (verb + conjunction + verb) to code *causal*]. This seemingly higher syntactic complexity provides further explanations to their acquisition in this later stage of early childhood.

Finally, the semantic content categories of *recurrence*, *notice*, *adversative*, *epistemic*, *specification*, and *communication* were found not to be acquired by the 4-year-old group, using the 90% criterion. Once again, the late acquisition of the above semantic content categories, except *recurrence*, aligns the order suggested by Stockman and Vaughn-Cooke (1986) and Bloom (1991). From the cognitive perspective, as noted in previous section, an increment in the cumulative cognitive complexity among *additive*, *temporal*, *causal*, and *adversative* is suggested. The content of *adversative* does not only represent definite events with highest cumulative cognitive complexity, but additionally denotes complex contrastive relationship beyond the here-and-now context (Bloom, 1991). The highest cognitive complexity of *adversative* thus explained its later acquisition. On the other hand, it is also proposed that the semantic content categories about how people think (*epistemic*) or talk (*communication*) about events, which involve implicit and non-transparent reasoning processes, may be complex for young children to interpret because it is considered relatively difficult for them to take the perspective of others (Evers-Vermeul and Sanders, 2011). In addition, some specific lexicons are also required in representing the definite relations of events in certain semantic content categories (e.g., perceptual verb to code content of *notice*; mental state verb to code content of *epistemic*; verb for conversation to describe what is to be express in the content of *communication*). The challenges in acquiring these specific and abstract words therefore pose extra difficulties in acquiring the corresponding semantic content categories.

Syntactically, in Mandarin, expressions of *adversative* usually require the use of conjunctions between clauses, and generally appear after those associated with simple sentences, but earlier than those related with more complex complementation (i.e., *epistemic*, *notice*, *communication).* Semantic content categories related with complementation, subsequently, also appeared earlier than those related to relativization (i.e., *specification*). The higher syntactic complexity of these semantic content categories may therefore explain why they are not acquired by the 4-year-old children.

It is noted that the semantic content category of *specification* was absent at all ages in the current study, which is coherent with the infrequent occurrence in young children reported in Bloom (1991). According to Lahey (1988), *specification* is usually expressed to indicate a particular person, object or event in terms of their functions, places or activities. More specific relational knowledge is thus required for its production. In addition, expression of *specification* usually involves complex sentences with relativization (e.g., 穿红裙子的女孩在哭) (Bloom, 1991). According to Arndt and Schuele (2013), typically developing children usually start to form sentences containing relative clauses between the ages of 4 and 5 years of age and they continue to master the productions through the school-age year. It is therefore believed that this semantic content category only emerged at an older age, probably after age of five due to higher demand in cognitive ability and syntactic knowledge.

Unexpectedly, the semantic content category of *recurrence* was not acquired by the age of five in the current study. Likewise, it is also interesting to observe that the occurrence of the early acquired semantic content category of *reject* declined across ages and its occurrence did not reach 90% after the age of three. Both contents represent knowledge of single object and event and possess relatively low cognitive complexity. Syntactically, both can be encoded with simple phrases [e.g., 再吃 (adverb + verb) to code *recurrence*; 不玩 (negative marker + verb) to code *reject*]. With relatively simple cognitive complexity and syntactic structure, these contents are expected to be acquired early. One possible reason is that the provided communicative context may not be obligatory enough to elicit the content categories of *recurrence* and *reject*. In addition, the high occurrence of *reject* in the 2-year-old group may also be related to the stage of “trouble two” in children’s development. It has been proposed that frequent non-compliance is common in toddlerhood and peaks during the second year (Tremblay, 2004; Alink et al., 2006). Most children then learn to regulate their behavior as they grow into preschool years and their non-compliance declines (Tremblay, 2003). Our findings appear to be consistent with this pattern of child development. On the other hand, it is also possible that some cultural specific factors may affect the production of these semantic content categories. Accordingly, social harmony and fitting in with others are more encouraged in the Chinese context than in the Western ones (Wu, 1996). According to Xu and Farver (2009), Chinese children tend to exhibit the regulated shyness, as a self-controlled form of social restraint to maintain harmonious group functioning and exemplifies self-regulation. Since *reject* and *recurrence* are mostly expressed when children intended to refuse or make additional request on objects or events, these may not adhere the social harmony in the Chinese culture. Similarly, it is also suggested that Chinese children are instructed to be more obedient due to the influence of Confucian teachings in traditional Chinese culture (Xiao, 1999). Older children may therefore tend to produce fewer negative expressions to reject, especially when interacting with our examiners that are all adults during the data collection. Considering all of the above, young Mandarin-speaking children therefore appeared to express fewer *recurrence* and *reject* contents in the current study.

To conclude, the acquisition trajectory of semantic content category in Mandarin-speaking-children aligns the sequence suggested by Bloom (1991) for English-speaking children. Acquisitions in both languages are predominantly predicted by the associated cognitive and syntactic complexity, while specific linguistic properties in Mandarin and Chinese culture also seem to have a role in modulating the acquisition of particular semantic content categories.

### Potential usage of the database in investigating the form-content interface in language acquisition of young children

According to Bloom (1991), young children acquire syntax and semantics together to lay the foundations of early expressions, and these two domains interact in a bidirectional manner to regulate the acquisition in early child language (Mok and Kipka, 2009). As mentioned, a certain form can be used to express various semantic content categories while an individual semantic content category can also be expressed with different forms. On some occasions, children who fail to encode the concept with specific grammatical forms, may still be able to possess the semantic content category with the expressions of the non-standard or ungrammatical forms (e.g., 爸爸鞋鞋 “daddy shoe” with absence of the genitive marker “*de*” to express *possession*). In view of the above, the importance to consider both language form and semantic content, as well as their interaction in child language study should not be underestimated.

The current study adopted language sample analysis, which allows the elicitation of various semantic content categories even among the youngest children by using standardized protocol. The established CMCL does not only provide important information on the acquisition of various semantic content categories in Mandarin-speaking children, but allows further investigation on the interaction between language form and content. By analyzing language samples annotated both syntactically (with parts of speech) and semantically (with semantic content categories), the relations between form and content produced by Mandarin-speaking preschoolers were further investigated. In the following, the syntactic category of verb and the semantic content category of *dative* were used as examples to illustrate the usage of the database to explore the interaction between form and content in the language acquisition of Mandarin-speaking children.

### Expression of verb (form) with different semantic content categories

In the past, vast amount of research studies have been conducted to investigate the acquisition of early vocabulary by young children but conflicting results in the acquisition of nouns and verbs in early child language have been documented in the literature. Gentner (1982) proposed that nouns are universally acquired before verbs, while the others found that verbs can also appear in children’s earliest vocabularies (Ma et al., 2009). Besides, Tardif (1996) also argued that young Mandarin-speaking children actually produced more verbs than nouns in their naturalistic speech. In view of this, the different semantic content categories encoded using verbs in the CMCL were explored in the current study. By searching utterances annotated with the part of speech “verb” in the CMCL, it is found that verb was used to express semantic content category of *action*, *state*, *locative action*, *dative*, *notice*, *communication*, and *epistemic*. Consider the proposed order of acquisition, *action* and *state* were the earliest semantic content categories to be acquired among all. *Locative action* and *dative* followed these two and were acquired by the 3-year-old and 4-year old children, respectively. Finally, the content of *notice*, *communication*, and *epistemic* were not acquired till the age of five. As such, it will not be adequate to look at the surface form of the vocabulary alone, but to examine different semantic content categories being expressed by the form, in order to have a more comprehensive picture on early language acquisition. By classifying verb according to the different semantic content categories being represented, it is possible to explain why some verbs appear earlier in age whereas the others emerge later. It appeared that the acquisition of different lexical forms in young children is affected by the semantic content being encoded. With the syntactically and semantically annotated database, it is possible to investigate the form-content interface more thoroughly.

### Expression of dative (semantic content category) with different forms

Next, the expressions of the semantic content category of *dative* with different forms were investigated. Utterances expressing the content of *dative* designate the recipient of an object or action with or without a preposition (Lahey, 1988). These utterances were extracted from the CMCL for further investigation of different lexical items and sentence structures produced by participants across ages. The lists of lexical items produced were shown in Table 9.

**TABLE 9:** Lexical items expressing dative content produced by each age group.

Age group	Lexical items expressing dative content
1	给_1_^i^, 给_2_^ii^, 给_3_^iii^, 帮
2	给_1_^i^, 给_2_^ii^, 给_3_^iii^, 帮, 喂, 陪, 让, 请
3	给_1_^i^, 给_2_^ii^, 给_3_^iii^, 帮, 喂, 陪, 让, 请, 送, 对, 给

i. 给_1_ serves as a verb (e.g., 给我一本书).

ii. 给_2_ serves as a preverbal preposition (e.g., 给他送一本书).

iii. 给_3_ serves as a post verbal preposition (e.g., 送给他一本书 or 送一本书给他) (Li and Thompson, 1981).

The repertoire of lexical items expressing *dative* content was observed to be expanding across age groups. Older children were more capable in using a larger variety of lexical items, including verbs and prepositions, to express the content of *dative*. Considering the syntactic structures, it was found that the 2 year olds were able to produce sentences with subject-predicate (e.g., 我给它这个), prepositional phrase (e.g., 给她穿上衣服) and serial verb construction (e.g., 奶奶帮它刷) in expressing the content of *dative*. On the other hand, the older children were capable in using pivotal construction (e.g., 我们要请你吃饭) in addition to the above sentence types. In addition to earlier findings that the content of *dative* was only fully acquired by children in the oldest age group, these results indicate that older children were able to express the content with a larger variety of lexical items and syntactic structures.

From the above examples, by searching utterances annotated with verbs, it is possible to examine different semantic content categories being expressed. On the other hand, by searching utterances annotated with *dative* content, different lexical items and syntactic structures produced by participants across age groups can also be analyzed. Thus, the CMCL provides a useful and convenient tool for us to study the language acquisition of young children, from both syntactic and semantic perspectives. A preliminary platform for investigating this interaction between syntactic form and semantic content in the language acquisition process of Mandarin-speaking children is provided. It is further proposed that children acquire language in a two-dimensional way with various form-content interactions. When acquiring a particular form, children progressively broaden the semantic content categories being expressed by that form. Similarly, when acquiring a particular semantic content category, children also gradually expand the use of different lexical items and syntactic structures to represent that semantic content category.

In this study, the CMCL that documented the production of both content (semantic content category) and form (part of speech) was established. By analyzing data extracted from the database, our findings do not only provide insights in the acquisition trajectory of semantic content category in Mandarin-speaking children, but also shed new light on the understanding of language acquisition in the semantic domains. Apart from the influence of cognitive and syntactic complexity, specific language properties in Mandarin and Chinese culture also seem to play a role in the acquisition. More in-depth investigation on the acquisition of particular semantic content category in Mandarin is therefore inspired and supported by our preliminary findings. The CMCL will also be interesting for the broader research community and allow further research including the investigation of the form-content interface in the early language of children. It may further contribute to validate different theories in child language acquisition. Clinically, our findings inspire a potentially more comprehensive approach in profiling the language ability of children with typical development, as well as those with language disorders. Assessment and intervention for children with language disorders can be planned with a balanced consideration between form and content.

## Limitations and future directions

Our results present an initial acquisition order on various semantic content categories in Mandarin-speaking children, and support that same set of factors, i.e., syntactic and cognitive complexity, predominantly affect the acquisition of semantic content categories in Mandarin. Nevertheless, some semantic content categories such as *specification* and *epistemic* may be too complex and abstract to acquire at an early age (Bloom, 1991; Evers-Vermeul and Sanders, 2011). Future research should therefore be extended to include older children, so as to gain a clear overall picture on the usage and acquisition of various semantic content categories. On the other hand, LSA provides a rich communicative context and allows naturalistic language production in children (Evans and Craig, 1992; Owens, 2010), but some cultural-sensitive semantic content category such as *recurrence* and *reject* may not be elicited within the context provided. It may be necessary in future studies to include some structural language-eliciting procedures, to capture some particular semantic content categories. Finally, other limitations of the current study included its cross-sectional nature and the uneven number of subjects in each age group. More participants with a more even distribution of subjects across ages, as well as longitudinal data, would be preferable in future study.

## Conclusion

The current study reported a preliminary result on language acquisition in Mandarin-speaking children using a syntactically and semantically annotated database—CMCL. The replication of findings on sentence complexity and lexical diversity in typical language acquisition confirms the quality of the language sample data in the corpus for studying child language acquisition. Coherent findings in the acquisition of semantic content categories also appear to suggest that the acquisition trajectory of semantic content categories in Mandarin-speaking children mostly resembles that of English-speaking peers. Notably, the acquisition trend is largely explained by the cognitive and syntactic complexity, with additional influence from the language specific properties and cultural specific factors in Mandarin. In addition, with the tags in both part-of-speech and semantic content category, the CMCL does not only contributes additional perspective in studying child language from the semantic domain, but provides a useful platform for examining the interaction between language form and semantic content in early child language acquisition, which also implies significantly on the theoretical and clinical ground.

## Data availability statement

The raw data supporting the conclusions of this article will be made available by the authors, without undue reservation.

## Ethics statement

The studies involving humans were approved by The Human Subjects Ethics Sub-Committee of The Hong Kong Polytechnic University (Reference Number: HSEARS20191004001). The studies were conducted in accordance with the local legislation and institutional requirements. Written informed consent for participation in this study was provided by the participants’ legal guardians/next of kin.

## Author contributions

TT and DL contributed to the conception and design of the study, discussed the results, and contributed to the final manuscript. DL and M-TL supervised the whole project. TT conducted the experiment, organized the database and performed the statistical analysis, and wrote the first draft of the manuscript. All authors contributed to manuscript revision, read, and approved the submitted version.
