# Corrigendum: Corpus of Mandarin Child Language: a preliminary study on the acquisition of semantic content categories in Mandarin-speaking preschoolers

**DOI:** 10.3389/fpsyg.2024.1359920

**Published:** 2024-01-16

**Authors:** Tempo Po-Yi Tang, Dustin Kai-Yan Lau, Man-Tak Leung

**Affiliations:** Department of Chinese and Bilingual Studies, The Hong Kong Polytechnic University, Kowloon, Hong Kong SAR, China

**Keywords:** semantic content category, language corpus, Mandarin-speaking children, cognitive and syntactic complexity, acquisition

In the published article, there were errors in Table 8 as published. The line indicating the percentage of the occurrence of different semantic content categories for Reject, Denial, Additive, Recurrence and Adversative was incorrect. The corrected Table 8 appears below.

**Table 8:** Occurrence of different semantic content categories by age group.

	**2 year olds**	**3 year olds**	**4 year olds**
Existence	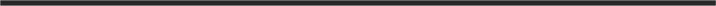		
Nonexistence	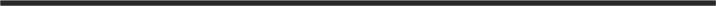		
Attribution	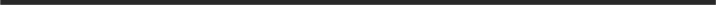		
Action	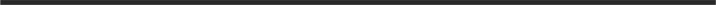		
Locative state	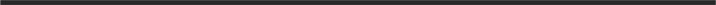		
State	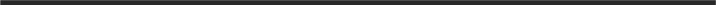		
Quantity	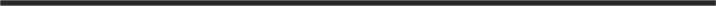		
Temporal	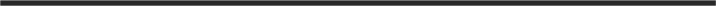		
Reject	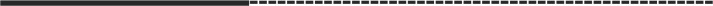		
Denial	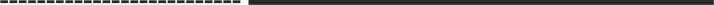		
Locative action	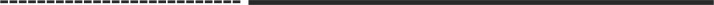		
Possession	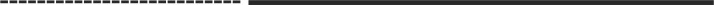		
Additive	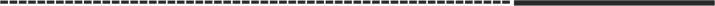		
Causal	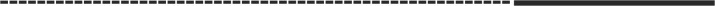		
Dative	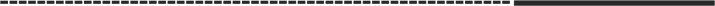		
Recurrence	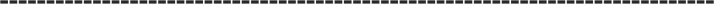		
Notice	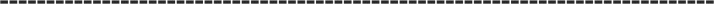		
Adversative	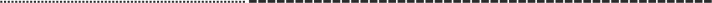		
Epistemic	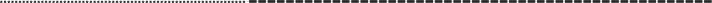		
Communication	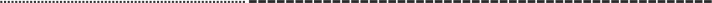		
Specification	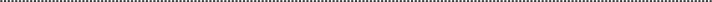		

A dotted line indicates that the semantic content category had < 20% occurrence, a dashed line indicates 20%−89% occurrence, and a solid line indicates ≥90% occurrence.

The authors apologize for this error and state that this does not change the scientific conclusions of the article in any way. The original article has been updated.

