# Supplementary material for: Corpus of Mandarin Child Language: a preliminary study on the acquisition of semantic content categories in Mandarin-speaking preschoolers
Source: Front Psychol. 2023 Nov 10;14:1234525. doi: 10.3389/fpsyg.2023.1234525 (PMC10667479; doi:10.3389/fpsyg.2023.1234525)
Supplement: Supplementary file 1 [file Table_1.docx]

Supplementary Table 1. Average token count of 21 semantic content categories across age groups

|  | **Age group 1** | | **Age group 2** | | **Age group 3** | |
| --- | --- | --- | --- | --- | --- | --- |
|  | Mean | S.D. | Mean | S.D. | Mean | S.D. |
| Existence | 108.63 | 41.97 | 136.37 | 52.46 | 118.59 | 54.29 |
| Recurrence | 3.11 | 4.92 | 2.41 | 2.55 | 1.50 | 2.15 |
| Nonexistence | 6.16 | 2.83 | 6.90 | 5.65 | 3.36 | 2.46 |
| Rejection | 3.11 | 2.62 | 3.32 | 4.03 | 1.14 | 1.75 |
| Denial | 9.42 | 6.85 | 13.51 | 5.41 | 12.68 | 4.99 |
| Attribution | 31.58 | 20.27 | 62.39 | 28.74 | 60.82 | 37.66 |
| Possession | 4.53 | 4.31 | 6.05 | 4.98 | 8.91 | 7.23 |
| Locative Action | 4.11 | 3.21 | 8.24 | 6.44 | 5.50 | 5.15 |
| Action | 63.11 | 27.41 | 79.76 | 36.61 | 66.77 | 32.49 |
| Locative State | 11.89 | 7.48 | 12.20 | 6.08 | 11.23 | 5.94 |
| State | 15.32 | 8.87 | 21.88 | 12.38 | 21.59 | 12.20 |
| Quantity | 7.58 | 5.81 | 12.24 | 9.49 | 11.27 | 8.48 |
| Notice | 0.63 | 0.83 | 1.44 | 2.34 | 0.77 | 1.31 |
| Dative | 2.05 | 1.84 | 3.00 | 4.06 | 2.77 | 2.52 |
| Additive | 2.89 | 4.03 | 4.54 | 5.02 | 7.36 | 5.06 |
| Temporal | 7.89 | 6.75 | 19.15 | 16.81 | 18.27 | 14.65 |
| Causal | 1.26 | 1.73 | 2.85 | 3.29 | 3.86 | 5.44 |
| Adversative | 0.11 | 0.32 | 1.54 | 2.31 | 1.36 | 1.84 |
| Epistemic | 0.16 | 0.69 | 0.80 | 1.52 | 0.36 | 0.73 |
| Specification | 0.00 | 0.00 | 0.00 | 0.00 | 0.00 | 0.00 |
| Communication | 0.16 | 0.50 | 1.02 | 1.54 | 0.59 | 1.10 |
