# Supplementary material for: Corpus of Mandarin Child Language: a preliminary study on the acquisition of semantic content categories in Mandarin-speaking preschoolers
Source: Front Psychol. 2023 Nov 10;14:1234525. doi: 10.3389/fpsyg.2023.1234525 (PMC10667479; doi:10.3389/fpsyg.2023.1234525)
Supplement: Supplementary file 2 [file Table_2.docx]

Supplementary Table 2. Average number of different semantic content categories per utterance across age groups

|  | Age group 1 | | Age group 2 | | Age group 3 | |
| --- | --- | --- | --- | --- | --- | --- |
|  | Mean | S.D. | Mean | S.D. | Mean | S.D. |
| Existence | 0.838 | 0.214 | 0.990 | 0.279 | 1.051 | 0.228 |
| Recurrence | 0.025 | 0.036 | 0.017 | 0.017 | 0.012 | 0.014 |
| Nonexistence | 0.051 | 0.024 | 0.048 | 0.038 | 0.030 | 0.021 |
| Reject | 0.029 | 0.029 | 0.021 | 0.021 | 0.011 | 0.020 |
| Denial | 0.074 | 0.056 | 0.100 | 0.036 | 0.120 | 0.047 |
| Attribution | 0.240 | 0.136 | 0.458 | 0.188 | 0.536 | 0.234 |
| Possession | 0.037 | 0.033 | 0.044 | 0.035 | 0.083 | 0.066 |
| Locative Action | 0.029 | 0.021 | 0.058 | 0.040 | 0.047 | 0.034 |
| Action | 0.481 | 0.140 | 0.568 | 0.166 | 0.590 | 0.134 |
| Locative State | 0.088 | 0.051 | 0.089 | 0.041 | 0.102 | 0.050 |
| State | 0.031 | 0.016 | 0.039 | 0.019 | 0.049 | 0.021 |
| Quantity | 0.058 | 0.041 | 0.087 | 0.061 | 0.095 | 0.049 |
| Notice | 0.006 | 0.008 | 0.010 | 0.017 | 0.008 | 0.014 |
| Dative | 0.019 | 0.020 | 0.020 | 0.024 | 0.024 | 0.021 |
| Additive | 0.022 | 0.032 | 0.034 | 0.037 | 0.069 | 0.045 |
| Temporal | 0.064 | 0.051 | 0.136 | 0.113 | 0.161 | 0.101 |
| Causal | 0.010 | 0.014 | 0.019 | 0.020 | 0.032 | 0.036 |
| Adversative | 0.001 | 0.002 | 0.011 | 0.015 | 0.011 | 0.014 |
| Epistemic | 0.001 | 0.005 | 0.005 | 0.009 | 0.004 | 0.007 |
| Specification | 0.000 | 0.000 | 0.000 | 0.000 | 0.000 | 0.000 |
| Communication | 0.001 | 0.003 | 0.007 | 0.011 | 0.005 | 0.009 |
